# A VSD-based framework for assessing climate justice in urban outdoor cooling spaces: a case study of Fuzhou, China

**DOI:** 10.3389/fpubh.2025.1724719

**Published:** 2025-12-12

**Authors:** Fengxiao Cao, Yimeng Zhou, Yu Luo, Yuming Shang, Jinsu Yang, Di Yang

**Affiliations:** 1 College of Architecture and Planning, Fujian University of Technology, Fuzhou, China; 2 Key Laboratory of New Technology for Construction of Cities in Mountain Area, Ministry of Education, Chongqing University, Chongqing, China

**Keywords:** climate justice, heat vulnerability, urban outdoor cooling spaces, evaluation and optimization, VSD framework

## Abstract

**Introduction:**

Extreme heat events intensify health risks among vulnerable populations, raising concerns regarding climate justice. However, most existing assessments remain at the citywide scale and seldom examine inequities across different types of outdoor cooling spaces.

**Methods:**

This study integrates the Exposure–Sensitivity–Adaptive Capacity (VSD) framework with the dimensions of distributive, recognition, and procedural justice to construct a climate justice assessment model. Outdoor cooling spaces were classified into linear and areal forms. Using Gulou District in Fuzhou as the case study, we developed a multi-source indicator system based on remote sensing imagery, street-view data, points of interest, and demographic statistics. The entropy weight method was used to determine indicator weights, and K-means clustering was applied to identify climate injustice space types.

**Results:**

The findings show that 37.18% of linear cooling spaces and 44.45% of areal cooling spaces face significant climate injustice risks. High-risk areas are concentrated in dense built-up zones, aging neighborhoods, and peripheral areas with limited public services. Cluster analysis identified three distinct deficit categories: distributional justice deficit, recognitional justice deficit, and systemic justice deficit, reflecting overlapping vulnerabilities and uneven adaptive capacities.

**Discussion:**

These results highlight the need for differentiated interventions to reduce spatial inequities. Key actions include enhancing shading along traffic corridors, improving service accessibility in aging neighborhoods, and strengthening adaptive resources in systemic deficit areas. The proposed framework offers an evidence base for equity-oriented urban governance and supports resilient urban planning and public health strategies under extreme heat.

## 1 Introduction

With global climate warming and rapid urbanization, extreme heat events are becoming more frequent and intense in cities worldwide (1). Such climatic extremes have been shown to influence not only ecological systems but also economic performance (2), posing serious challenges to the health and well-being of urban residents. Problems such as surface hardening, restricted ventilation, and a lack of green space intensify the urban heat island effect. As a result, urban residents, especially the old adult, outdoor workers, and low-income groups, face concentrated heat exposure and heightened health risks (3). Urban outdoor cooling spaces play a critical role in providing short-term thermal relief and rest under high-temperature conditions. The quality of their spatial supply and the equity of their services have become focal issues in adaptive urban planning and climate justice research (4). In this study, urban outdoor cooling spaces are defined as publicly accessible open spaces with cooling and heat-mitigation functions during heatwaves. They mainly include three typical types: streets, squares, and parks. These spaces operate at a micro-scale to serve daily resident needs. They typically reduce heat exposure risks and improve outdoor thermal comfort through vegetation shading, evaporative cooling from water bodies, ventilation enhancement, or the provision of supportive facilities.

Although climate justice has recently become an important value framework in environmental policy and climate adaptation research (5, 6), its assessment in relation to urban cooling spaces remains underdeveloped. Both the theoretical system and empirical approaches still need to be established. Recent studies have overlaid spatial patterns of heat exposure with the distribution of socially vulnerable groups to identify areas of heat inequity, highlighting the social disparities in heat exposure (7). Some research has examined urban green spaces, parks, and public facilities such as restrooms to analyze unequal provision and service coverage gaps. These studies have proposed spatial optimization strategies from a distributive justice perspective (7–9). Other scholars have focused on the participatory rights of disadvantaged groups in spatial governance, proposing ways to integrate procedural justice and participatory mechanisms into adaptive urban planning (10–12), while recent research has also emphasized the importance of institutional collaboration and social identity in achieving equitable adaptation (13, 14). Overall, current climate justice research remains concentrated on national- or city-level policy responses and adaptive capacity assessments (15–17). A comprehensive, multi-dimensional assessment framework is still lacking. Such a framework should integrate spatial resource allocation, population vulnerability, and service accessibility to fully reveal climate justice deficits at the micro scale (18, 19).

Current research on heat vulnerability assessment is continuously advancing. A clear trend is the shift from macro-level risk identification to fine-grained analysis at the micro spatial scale of cities (20–22). Most studies are based on the IPCC framework of “exposure–sensitivity–adaptive capacity.” They often build composite indicator systems, such as the Heat Vulnerability Index (HVI), to quantify risks (23, 24). Research methods have also extended from the macro scale to urban micro spaces. By integrating remotely sensed land surface temperature, points-of-interest (POI) data, and mobile phone signaling, scholars have spatialized key elements such as heat exposure, population distribution, and accessibility of medical resources (25–28). Among these approaches, the Vulnerability Scoping Diagram (VSD) framework has become a core method in urban heat vulnerability analysis due to its clear structure and strong operability (29, 30). However, most existing studies still emphasize risk identification. They pay insufficient attention to justice disparities underlying the risks (31, 32). Analytical approaches that integrate the heat vulnerability framework with concepts of social equity remain underdeveloped. As a result, current assessments provide limited support for the design of equity-oriented spatial intervention strategies.

To address the above research gap, this study integrates the VSD framework with climate justice theory. It links the “exposure–sensitivity–adaptive capacity” analysis with the dimensions of distributive, recognition, and procedural justice. This integration reframes vulnerability assessment from a descriptive risk identification tool into a normative framework for diagnosing spatial inequity. Focusing on micro-scale urban outdoor cooling spaces in Fuzhou’s Gulou District, the study develops tailored indicator systems for both linear and areal spaces. It then applies GIS-based clustering to reveal typical justice-deficit patterns. Theoretically, it extends the VSD framework toward a justice-oriented paradigm that bridges vulnerability research and climate equity theory. Methodologically, it operationalizes abstract justice concepts through measurable spatial indicators. Practically, it provides a replicable pathway for equity-oriented adaptive planning and governance under intensifying urban heat.

## 2 Methodology

### 2.1 Study area

**Figure 1:**
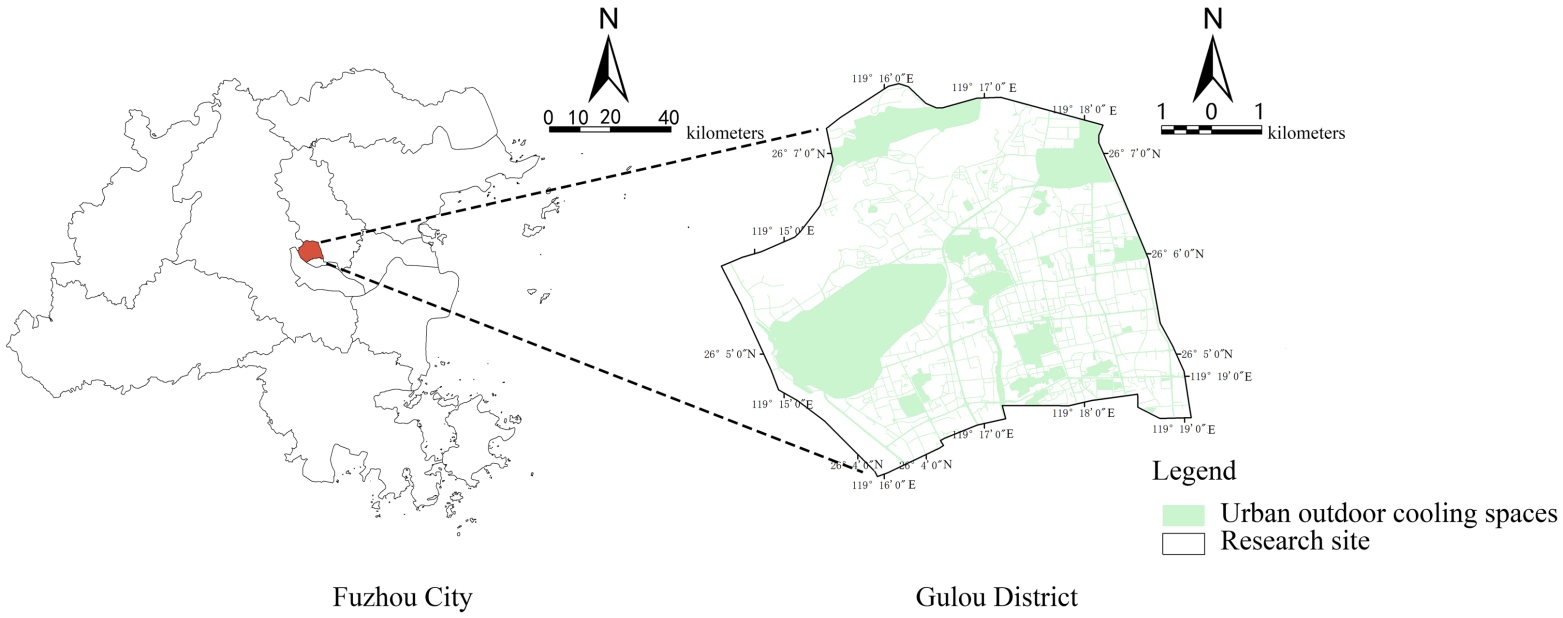
Study area.

Gulou District of Fuzhou was selected as the case study area (Figure 1). Fuzhou is located on the southeast coast of China and serves as the capital of Fujian Province. Gulou District lies at the city center and is the political, economic, and cultural hub of the city. The district has a population of approximately 678,000, with a density of 19,635 persons per square kilometer. Fuzhou is one of China’s four “furnace cities,” where extreme summer heat occurs frequently. The city faces severe urban thermal environment problems, providing a typical climatic context for this study. As the core built-up area of Fuzhou, Gulou District is characterized by dense building development, impervious surfaces, and limited green space. These factors jointly contribute to a strong urban heat island effect, making it a representative area for heat exposure risk studies. Nationally, Fuzhou represents the dual pressures of high thermal exposure and uneven adaptive capacity common to many Chinese cities undergoing old-city renewal. Internationally, its humid subtropical climate, coastal setting, and mixed socio-spatial structure resemble those of rapidly growing cities in the Global South, such as Bangkok and Ho Chi Minh City. The coexistence of high-end developments and aging neighborhoods within Gulou provides an ideal micro-scale setting for examining spatial inequities and adaptive governance under intensifying heat.

### 2.2 Data sources

**Figure 2:**
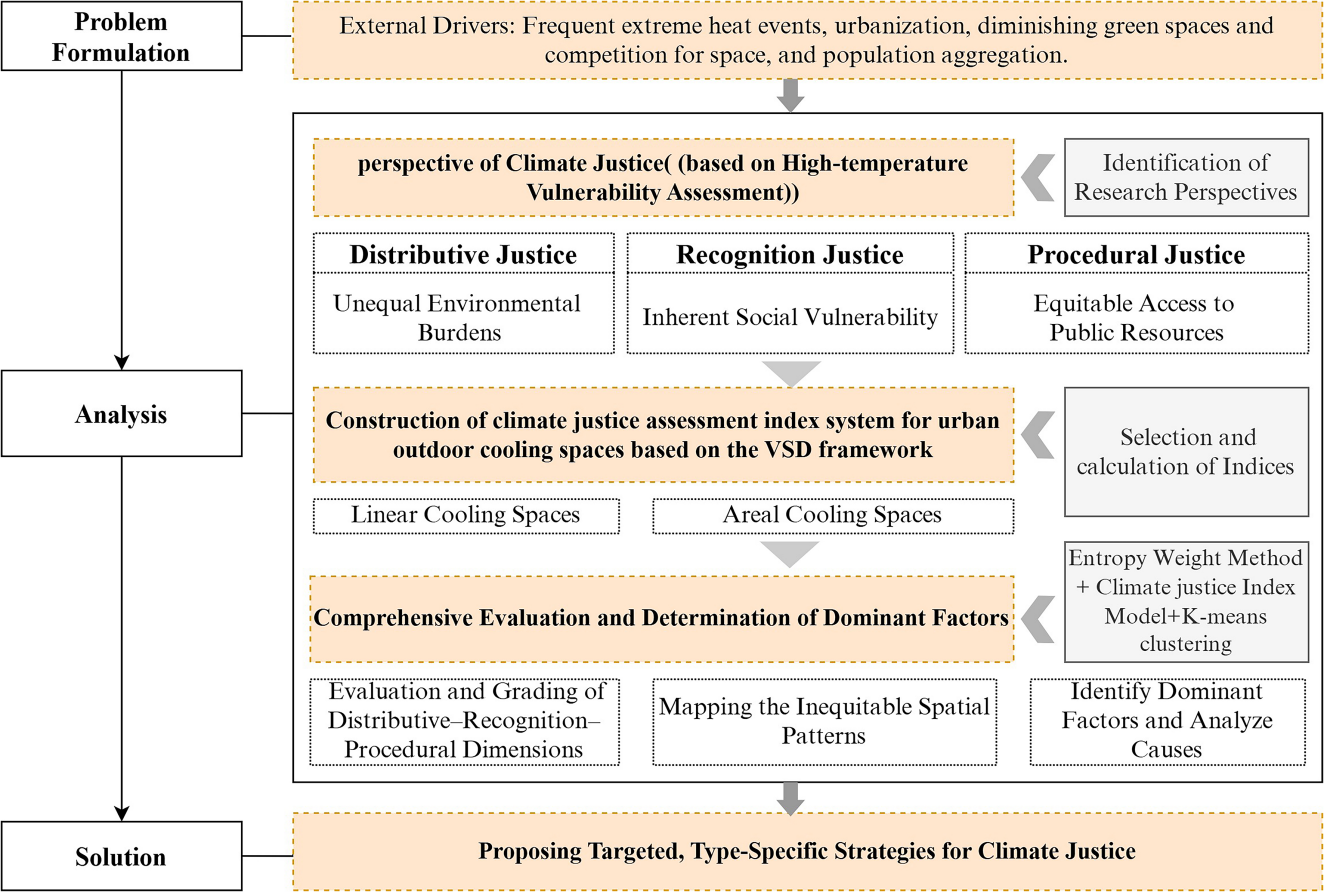
A research framework for this study.

This study quantifies climate justice in urban outdoor cooling spaces using multiple data sources (Table 1). The research framework is shown in Figure 2. First, drawing on the VSD framework and a climate justice perspective, this study translates the tri-dimensional logic of vulnerability (comprising exposure, sensitivity, and adaptive capacity) into three evaluation dimensions: distributive, recognition, and procedural justice. On this basis, an indicator system for assessing climate justice in urban outdoor cooling spaces was established. The indicators were calculated using the ArcGIS visualization platform. Second, the entropy weight method and functional model approach were applied to generate a spatial distribution map of climate justice in urban outdoor cooling spaces in Gulou District, Fuzhou. Finally, by identifying dominant risk factors, the study proposes management measures for addressing high-temperature risks in urban outdoor cooling spaces.

**Table 1:** The data sources.

Data type	Data content	Data source and annotation
Geospatial data	Street View panoramic images	Baidu Maps. Used for calculating Sky View Factor, Green View Index, etc., via machine learning semantic segmentation.
	Satellite remote sensing data	Landsat/Sentinel satellites. 10-meter resolution data used to calculate NDVI for vegetation coverage assessment.
	Surface Temperature	Landsat 8/9 Thermal Infrared Sensor (TIRS). Used to directly measure the heat load on different surfaces.
	Administrative boundaries, road network	OpenStreetMap. Used for defining the study area and spatial analysis framework.
Socioeconomic data	Point of Interest (POI)	Baidu Maps Database. Used Python web scraping techniques to obtain locations of medical facilities and indoor cooling facilities.
	Population Aggregation	Baidu Heat Map. Used to represent the spatial density of population activity.
	Housing Prices	Public real estate platforms. Used as a proxy indicator for the socio-economic status of neighborhoods.
	Sensitive Population	National Population Census data. Used to determine the proportion of sensitive groups such as the old adults and children.

The data used in this study include: (1) street-view panoramas obtained from Baidu Maps API (collected in August 2023) for calculating the Sky View Factor and Green View Index; (2) Landsat 8/9 and Sentinel-2 satellite imagery (10 m resolution, July 2023) for deriving land-surface temperature and NDVI; (3) Point-of-Interest (POI) data from Baidu Maps API (collected in July 2023) for identifying indoor cooling and medical facilities; (4) Baidu Heat Map data (July 2023, hourly mean 15:00–16:00) for representing population aggregation; (5) housing-price data from Anjuke (July 2023) as a proxy for neighborhood socio-economic status; (6) demographic data from the National Population Census of China (2020) to determine the proportions of old adult and children representing sensitive populations; and (7) administrative boundaries and road networks from OpenStreetMap (accessed in 2023). All spatial datasets were processed and integrated in ArcGIS for coordinate unification, normalization, and consistency checking prior to analysis.

### 2.3 Construction of the index system

#### 2.3.1 Construction of the index system

Climate justice theory critiques the disproportionate negative impacts of climate change on socially vulnerable groups and advocates for fairness in adaptation strategies (33). Vulnerability assessment frameworks serve as analytical tools of climate justice (25, 34). By systematically analyzing exposure, sensitivity, and adaptive capacity, this study identifies urban outdoor cooling spaces that are most vulnerable to climate hazards. The results provide a scientific basis for promoting equitable resource allocation and guiding policy interventions. The system considers climate justice in three core dimensions. First, distributive justice addresses the uneven spatial distribution of heat exposure. It emphasizes that thermal loads are not neutral natural phenomena but unequal environmental burdens shaped by both urban form and social structure. In this study, land surface temperature was selected as a direct quantitative indicator (35). Additional spatial morphological indicators, including sky view factor, street canyon ratio, green view index, and vegetation coverage, were used to reveal the physical drivers of high land surface temperatures (36–38). Second, recognition justice emphasizes the importance of acknowledging social diversity. It also calls for greater attention to the specific needs of vulnerable groups in policy-making. It reflects the inherent characteristics that make certain groups more susceptible to harm under the same heat exposure due to their socio-economic status and demographic attributes. Two dimensions were assessed in this study. Housing prices were used as a proxy for socio-economic conditions, as they strongly correlate with household income, community resources, and social stratification (39). In addition, the proportion of sensitive populations and population aggregation were employed to capture the unequal risks of exposure caused by demographic vulnerability and uneven population distribution (40–42). The share of old adults and children effectively represents population vulnerability because these groups have lower thermoregulatory efficiency, higher metabolic sensitivity, and limited behavioral adaptability to heat (43). Compared to working-age adults, they spend more time in local outdoor environments. This leads to a greater reliance on public cooling facilities, which are often inequitably distributed across urban neighborhoods (44, 45). Finally, procedural justice concerns the fairness of access to adaptive resources and participation in decision-making processes. It stresses that all groups should enjoy equal rights in accessing cooling resources and emergency services. To quantify differences in opportunities for accessing indoor cooling facilities and emergency medical assistance, two indicators were selected: the number of nearby indoor cooling facilities and the number of nearby medical institutions. The spatial variation of these facilities directly reflects inequalities in adaptive capacity (46, 47). Details are shown in Table 2.

**Table 2:** Indices for climate justice assessment within the VSD assessment framework.

Assessment dimensions	Relevant interpretation	Selection of indices
Distributive justice	Evaluates the unequal spatial distribution of heat stress arising from disparities in the physical environment.	Land Surface Temperature (68), Sky View Factor, Street Canyon Ratio (69), Green View Index, Vegetation Coverage (68)
Recognition justice	Identifies population groups disproportionately exposed to heat-related risks due to socioeconomic status and demographic characteristics, and examines their spatial clustering.	Population Aggregation (40), Proportion of Sensitive Population (41), Housing Prices (39)
Procedural justice	Assesses community capacity to access and utilize public cooling facilities and healthcare services during heatwaves, reflecting fairness in the distribution of essential public resources.	Number of Indoor Cooling Facilities (46), Number of Medical Facilities (47)

#### 2.3.2 Comprehensive climate justice assessment

##### 2.3.2.1 CRITIC method for weight determination

This study employs the entropy weight method to determine the weights of indicators in the evaluation system for urban outdoor cooling spaces (Table 3). The Jenks natural breaks method is used to classify the comprehensive evaluation results and to identify the spatial distribution of climate justice across the study area. The entropy weight method relies on the statistical distribution of the indicator data, which minimizes the influence of subjective judgment in weight assignment. All raw data were standardized prior to the calculation of indicator weights. The quantitative assessment framework used in this study is formalized through Equations 1–7, which define the procedures for normalization, entropy-based weight calculation, criterion-layer index computation, and the construction of the Climate Justice Index.

**Table 3:** Index system for climate justice assessment.

Target layer	Space type	Guideline dimension	Representative indicators (with indicative weights)	Description
Assessment of climate justice in outdoor cooling spaces	Linear cooling spaces (A1)	Distributive justice (B1)	Sky View Factor (0.190); Street Canyon Ratio (0.550); Green View Index (0.131); Surface Temperature (0.129)	Indicators reflect physical exposure to heat and shading conditions along linear spaces.
		Recognition justice (B2)	Population Aggregation (0.187); Sensitive Population (0.527); Housing Prices (0.286)	Capture demographic and socioeconomic sensitivity to heat stress.
		Procedural justice (B3)	Indoor Cooling Facilities (0.623); Medical Facilities (0.377)	Represent accessibility and fairness in cooling and medical services.
	Areal cooling spaces (A2)	Distributive justice (B1)	Vegetation Coverage (0.648); Surface Temperature (0.352)	Represent ecological cooling capacity and thermal exposure.
		Recognition justice (B2)	Population Aggregation (0.390); Sensitive Population (0.399); Housing Prices (0.211)	Reflect social vulnerability around areal cooling spaces.
		Procedural justice (B3)	Indoor Cooling Facilities (0.533); Medical Facilities (0.467)	Measure equitable access to public facilities supporting heat adaptation.

The normalization formulas are expressed as follows:

Positive normalization:


(1) $$Y_{\mathrm{ij}}=(X_{\mathrm{ij}}-X_{\text{jmin}})/(X_{\text{jmax}}-X_{\text{jmin}})$$

Negative normalization:


(2) $$Y_{\mathrm{ij}}=(X_{\text{jmax}}-X_{\mathrm{ij}})/(X_{\text{jmax}}-X_{\text{jmin}})$$

Here, $X_{\mathrm{ij}}$, $X_{\text{jmax}}$, $X_{\text{jmin}}$ and $Y_{\mathrm{ij}}$ denote the original value, maximum, minimum, and normalized value of the vulnerability indicators, respectively; *i* = 1, 2…, *m*; *j* = 1, 2…, *n*.

The entropy weight method was then applied to derive indicator weights:


(3) $$P_{\mathrm{ij}}=Y_{\mathrm{ij}}/\sum_{i=1}^{m}Y_{\mathrm{ij}}$$

(4) $$e_{j}=-(1/\ln(m))\sum_{i=1}^{m}p_{\mathrm{ij}}\ln(p_{\mathrm{ij}})$$

(5) $$\omega_{j}=(1-e_{j})/\sum_{j=1}^{n}(1-e_{j})$$

Here, $P_{\mathrm{ij}}$ is the proportion of the *i*-th sample under the *j*-th indicator; $e_{j}$ represents the information entropy of indicator *j*; and $\omega_{j}$ is the weight assigned to indicator *j*.

After the weights were obtained, weighted scores for distributive justice, recognition justice, and procedural justice were calculated. These scores were integrated in GIS to generate spatial distribution maps for each dimension. The Jenks natural breaks method was then used to classify each dimension into five levels (low, lower, medium, higher, high), thereby revealing spatial patterns of climate justice.

##### 2.3.2.2 Modeling of the climate justice assessment system

To capture the interactions among distributive, recognition, and procedural justice, a functional model was developed to assess climate justice in urban outdoor cooling spaces (29). The index of each criterion layer was first calculated as:


(6) $$C_{k}=\sum_{j-1}^{n}Y_{\mathrm{ij}}\omega_{j}(i=1,,,2,,,\ldots ,,,m)$$

where $C_{k}$ denotes the index of each criterion layer, and the other variables are as previously defined.

The Climate Justice Index (CJI) was then constructed by integrating the three dimensions, as expressed by:


(7) $$\mathrm{CJ}I_{i}=\mathrm{\alpha D}J_{i}+\mathrm{\beta R}J_{i}+\mathrm{\gamma P}J_{i}$$

where $DJ_{i}$, $RJ_{i}$ and $PJ_{i}$ represent the scores of unit *i* for distributive, recognition, and procedural justice, respectively *_α_*, *β* and *γ* are their assigned weights. A higher CJI indicates a higher level of climate justice in the spatial unit.

##### 2.3.2.3 Identify the dominant risk factors

To further identify the dominant factors underlying different vulnerability patterns, the K-Means clustering algorithm was applied. All spatial units were grouped into several categories with similar characteristics based on their standardized scores in distributive justice, recognition justice, and procedural justice. Subsequently, analysis of variance (ANOVA) was employed to test whether the mean values of the three dimensions differ significantly across categories. By comparing the relative values of each category, the dominant factors that define and differentiate each climate justice pattern were identified.

## 3 Results

### 3.1 Quantitative values of assessment indices

**Figure 3:**
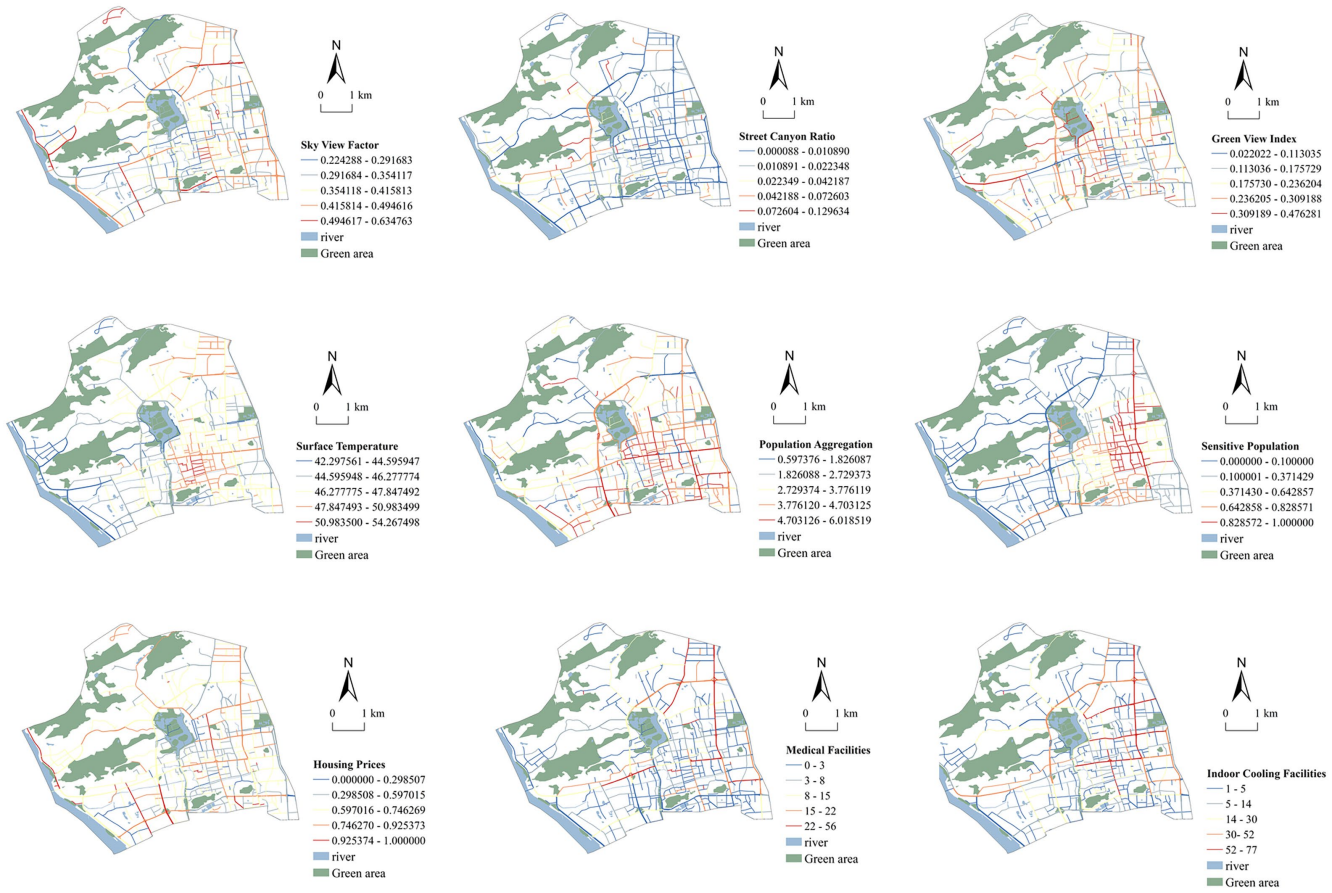
Quantitative values of climate justice assessment indices for linear cooling spaces.

**Figure 4:**
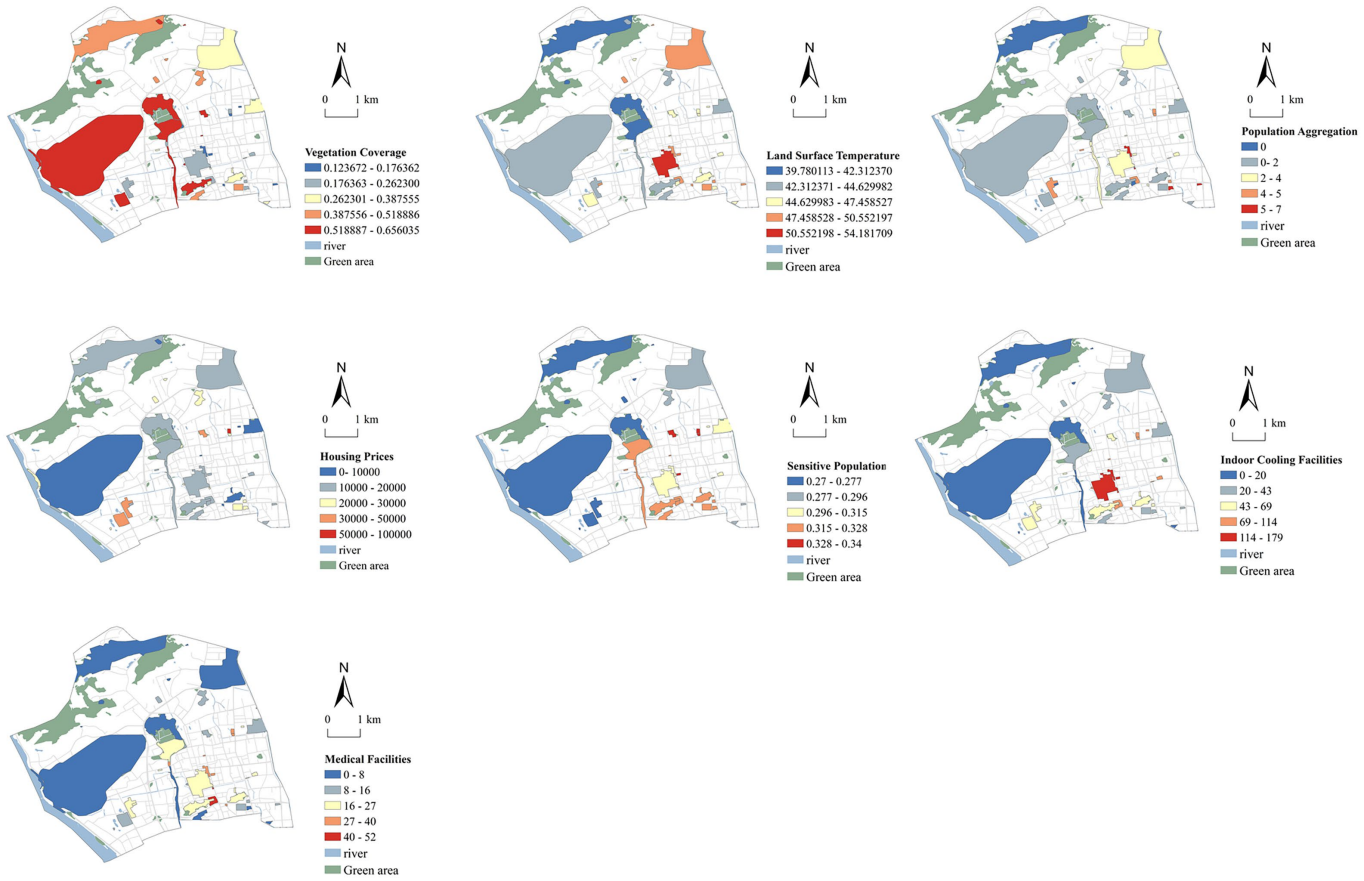
Quantitative values of areal cooling space climate justice assessment indices.

Figures 3, 4 illustrate the quantified results of 16 indicators for assessing climate justice in urban cooling spaces across Gulou District, Fuzhou. The patterns indicate marked spatial differences in thermal risk within the built environment. In linear cooling spaces, land surface temperature (C4) is generally higher in the central area and lower toward the periphery, with hotspots located along major roads, traffic-intensive zones, and streets lacking vegetation. The sky view factor (C1) and street enclosure (C2) display opposite patterns, as streets with lower SVF and higher enclosure tend to trap more heat. The green view index (C3) is most prominent in the northwestern part of the district and along several secondary roads, highlighting the cooling benefits of street vegetation. Population density (C5) and the proportion of vulnerable groups (C6) display similar spatial patterns, with high concentrations in traditional residential neighborhoods such as Dongjiekou, Jintai, and Nanhou Street. These areas face greater health risks during extreme heat events. Housing prices (C7) form high-value clusters around Dongjiekou and Wusi Road. This pattern reflects the relative advantage of these areas in adaptive capacity allocation, largely due to their accessibility to resources. The distribution of indoor cooling facilities (C8) and medical services (C9) declines gradually from the urban core toward the periphery. Central areas are well served, while peripheral streets show limited service coverage, revealing inequities in resource allocation.

In areal cooling spaces, land surface temperature (C11) is significantly higher in built-up areas that are distant from water bodies and green spaces. The most pronounced hotspots occur in the southwestern sector and several inland communities. In contrast, areas near Jinniu Mountain and Wenshan Park demonstrate stronger thermal regulation capacity, with relatively lower temperatures overall. Vegetation coverage (C10) shows a clear negative correlation with temperature, underscoring the role of high vegetation density in improving local thermal environments. Population aggregation (C12) and the proportion of sensitive populations (C13) are also high in old urban districts and along park boundaries, indicating a spatial mismatch between heat exposure and the availability of green resources. Housing prices (C14) follow a similar distribution pattern, with high-value areas concentrated in the eastern and southern parts of Gulou District, adjacent to traditional high-quality residential neighborhoods. Indoor cooling facilities (C15) and medical facilities (C16) are primarily concentrated in the central urban area. Notably, green-adjacent zones demonstrate significant deficiencies in service coverage and response capacity, revealing a spatial deficiency in procedural justice.

### 3.2 Spatial identification of climate injustice dimensions

#### 3.2.1 Distributive justice dimension

**Figure 5:**
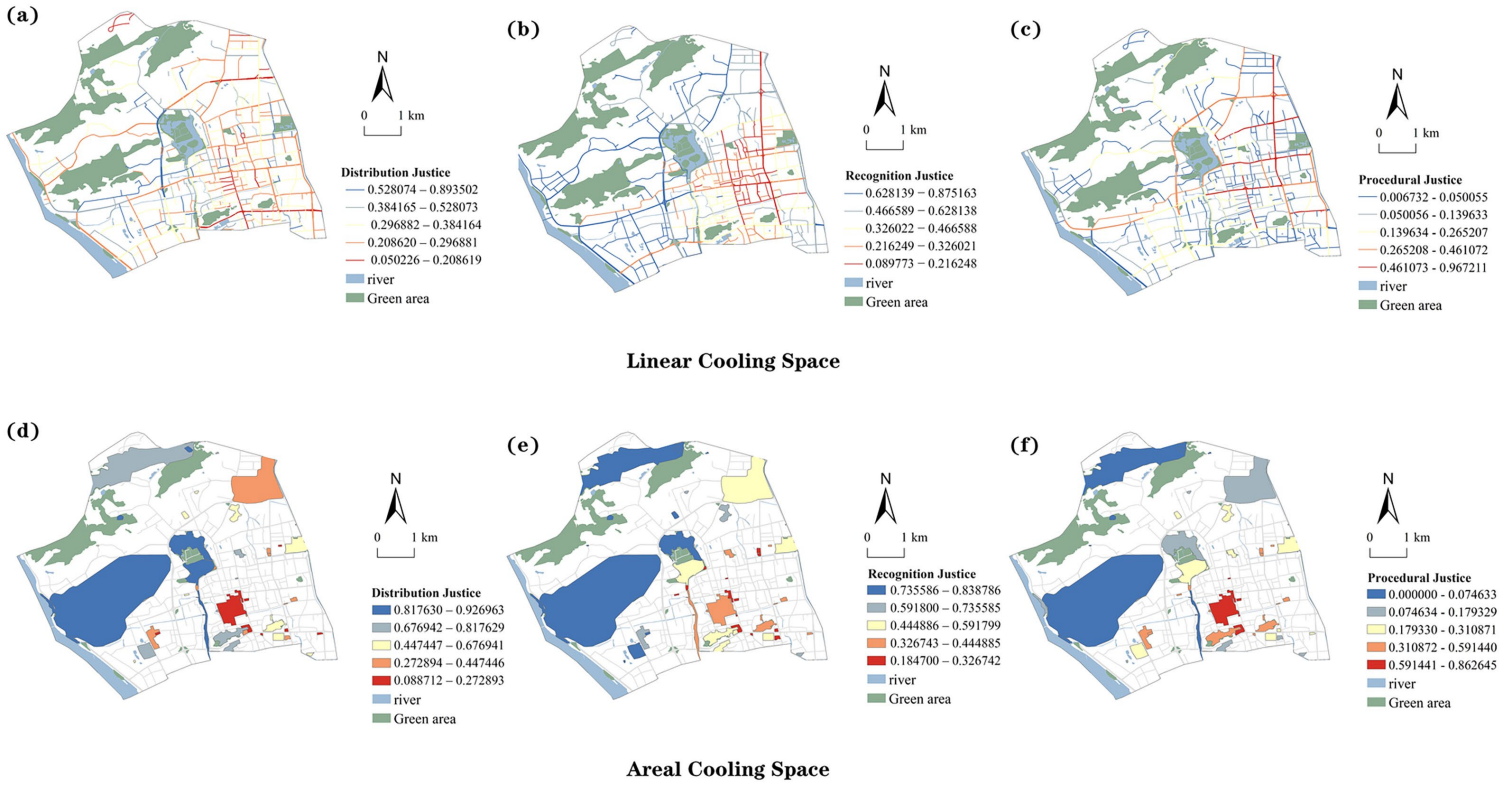
Distribution, recognition and procedural justice assessment classification. **(a)** Distributive justice in linear cooling spaces. **(b)** Recognition justice in linear cooling spaces. **(c)** Procedural justice in linear cooling spaces. **(d)** Distributive justice in areal cooling spaces. **(e)** Recognition justice in areal cooling spaces. **(f)** Procedural justice in areal cooling spaces.

Figure 5a and Table 4 present the assessment results of linear cooling spaces under the distributive justice dimension. Spaces with low and relatively low levels of distributive justice account for 38.48% of the total. These spaces are mainly distributed along major commuting corridors, including Baima Middle Road, the eastern section of Wusi Road, and the southern section of Hudong Road. These streets feature dense building enclosure, limited shading, and high vehicular heat emissions, functioning as primary channels for concentrated heat release. Targeted actions such as developing green corridors and installing microclimate regulation facilities are urgently needed in these zones.

**Table 4:** Proportion of level across dimensions for linear cooling spaces.

Level	Distribution justice	Recognition justice	Procedural justice
Low	6.83%	11.91%	52.69%
Lower	31.65%	20.58%	33.11%
Medium	34.53%	24.91%	9.32%
Higher	19.44%	21.30%	3.58%
High	7.55%	21.30%	4.30%

Figure 5d and Table 5 display the distributive justice results for areal cooling spaces. High heat exposure is concentrated in the southern and central built-up areas. Low-level spaces account for 17.31%, mainly clustered around Nanmendou Square, Wuyi Square, and the Wenshan Road intersection. Extensive paving, limited shading, and poor ventilation together intensify heat accumulation and radiation. Spaces with relatively low distributive justice levels account for 23.08%, extending into surrounding areas and constituting the core heat risk zones of planar spaces.

**Table 5:** Proportion of level across dimensions for areal cooling spaces.

Level	Distribution justice	Recognition justice	Procedural justice
Low	17.31%	24.07%	16.67%
Lower	23.08%	16.67%	22.22%
Medium	23.08%	14.81%	18.52%
Higher	21.15%	18.52%	27.78%
High	15.38%	29.63%	14.81%

#### 3.2.2 Recognition justice dimension

Figure 5b and Table 4 present the assessment results of linear cooling spaces under the recognition justice dimension. Spaces with recognition justice deficits account for 32.49%, forming a radial distribution pattern centered on the western old town and extending along major roads. Typical linear spaces with pronounced deficits include Wushan Road, Dadao Road, and the northern section of Gongye Road. These areas host a high concentration of aging residents whose demographic characteristics make them more vulnerable to heat stress. However, their specific needs have long been overlooked in spatial planning and policy resource allocation, reflecting a notable lack of social recognition.

Figure 5e and Table 5 display the results for areal cooling spaces. Recognition justice deficits are found in 29.63% of the areas, mainly concentrated in the Wushan district and older residential neighborhoods west of Bayiqi Road. These communities have dense populations and a large share of aging residents. Areas with relatively low recognition justice (18.52%) are distributed along the southeastern edge of West Lake Park and within several urban village settlements.

#### 3.2.3 Procedural justice dimension

Figure 5c and Table 4 present the procedural justice assessment of linear cooling spaces. The results reveal a clear spatial gradient, with higher values concentrated in the central areas and progressively lower values toward the periphery. Low-level procedural justice areas account for 52.69%, including Hualin Road, the southern section of Qunzhong Road, and Fufei North Road. These areas lack continuous green corridors and sufficient cooling facilities, suggesting that service provision has not effectively addressed accessibility and equity for vulnerable groups. This pattern reflects procedural shortcomings in policy implementation and resource allocation.

Figure 5f and Table 5 show the distribution of procedural justice in planar cooling spaces. Low-level spaces account for 16.67%, primarily located along the eastern side of Gongye Road, the northern section of Tongpan Road, and several small squares along Nantai Road. Facilities in these spaces are sparse, leaving residents with limited resources and restricted access to emergency support during heat events, which undermines the establishment of effective response mechanisms. In contrast, high-level areas (14.81%) are concentrated in resource-rich urban cores, highlighting the spatial imbalance in service distribution.

### 3.3 Comprehensive climate justice assessment and inequity patterns

**Figure 6:**
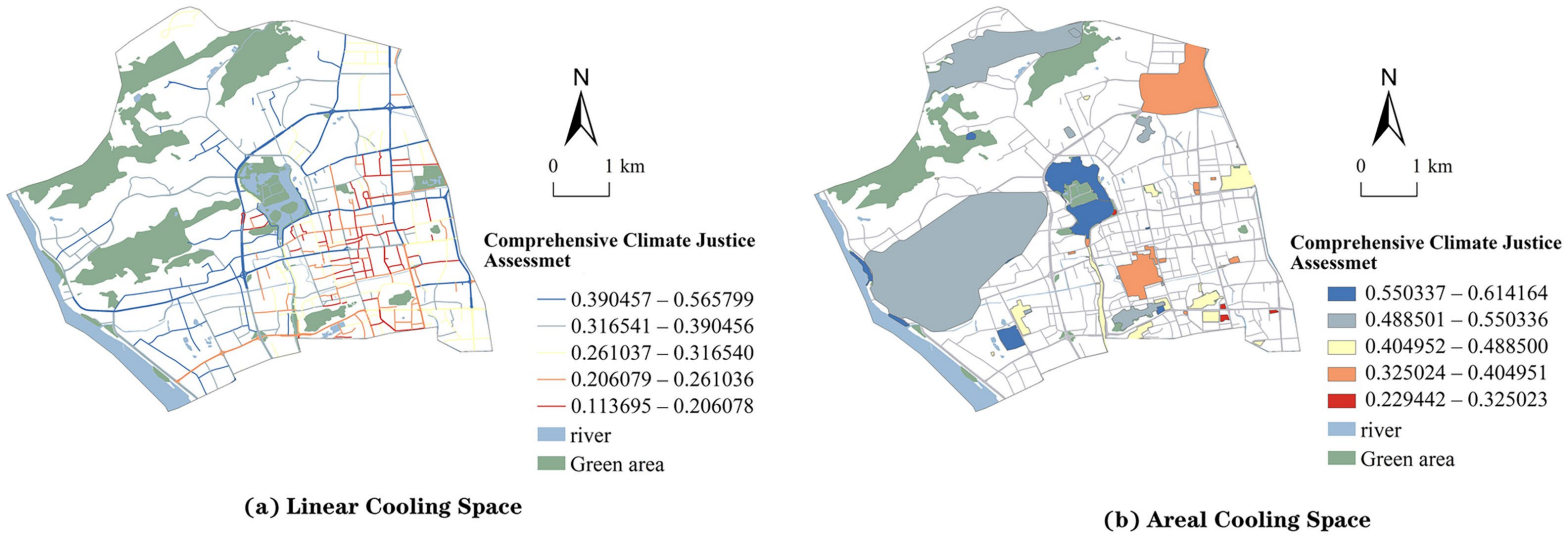
Comprehensive climate justice assessment. **(a)** Comprehensive climate justice in linear cooling spaces. **(b)** Comprehensive climate justice in areal cooling spaces.

Figure 6 illustrates the spatial distribution of climate justice in Gulou District based on the integrated assessment of distributive, recognition, and procedural justice. The overall level of climate justice in linear cooling spaces is relatively low (Figure 6a). Streets with low and lower justice levels account for 19.86 and 20.94%, mainly concentrated in the southern and southeastern parts of the district and in areas with dense arterial roads. These zones are adjacent to major traffic corridors, densely populated neighborhoods, and commercial clusters, where environmental burdens are high and vegetation and shading are relatively insufficient. In contrast, streets with high and higher climate justice levels are mainly distributed in the northwestern part and along ecological boundaries such as Jiangbin Road, accounting for 22.38 and 14.8%, respectively. These areas demonstrate stronger ecological buffering capacity (Table 6).

**Table 6:** Comprehensive evaluation statistics for linear cooling spaces.

Level	Count	Percentage
Low	41	14.8%
Lower	62	22.38%
Medium	61	22.02%
Higher	58	20.94%
High	55	19.86%

In areal cooling spaces, the assessment reveals marked variations in climate justice levels (Figure 6b). The results show that approximately 31.48% of these areas face serious climate justice issues, concentrated in the central and eastern built-up zones of the city, including the Dongjiekou commercial district and the surroundings of Wuyi Square. These areas are characterized by high population density, limited green coverage, and insufficient public services. Conversely, about 44.45% of the areas exhibit relatively high levels of climate justice. These are concentrated along ecological corridors and green systems at the urban periphery, such as Jinniu Mountain, Wushan, and Zuohai park. Such areas possess strong environmental carrying capacity and spatial adaptability to extreme heat events (Table 7).

**Table 7:** Comprehensive evaluation statistics for areal cooling spaces.

Level	Count	Percentage
Low	10	18.52%
Lower	14	25.93%
Medium	13	24.07%
Higher	13	24.07%
High	4	7.41%

**Figure 7:**
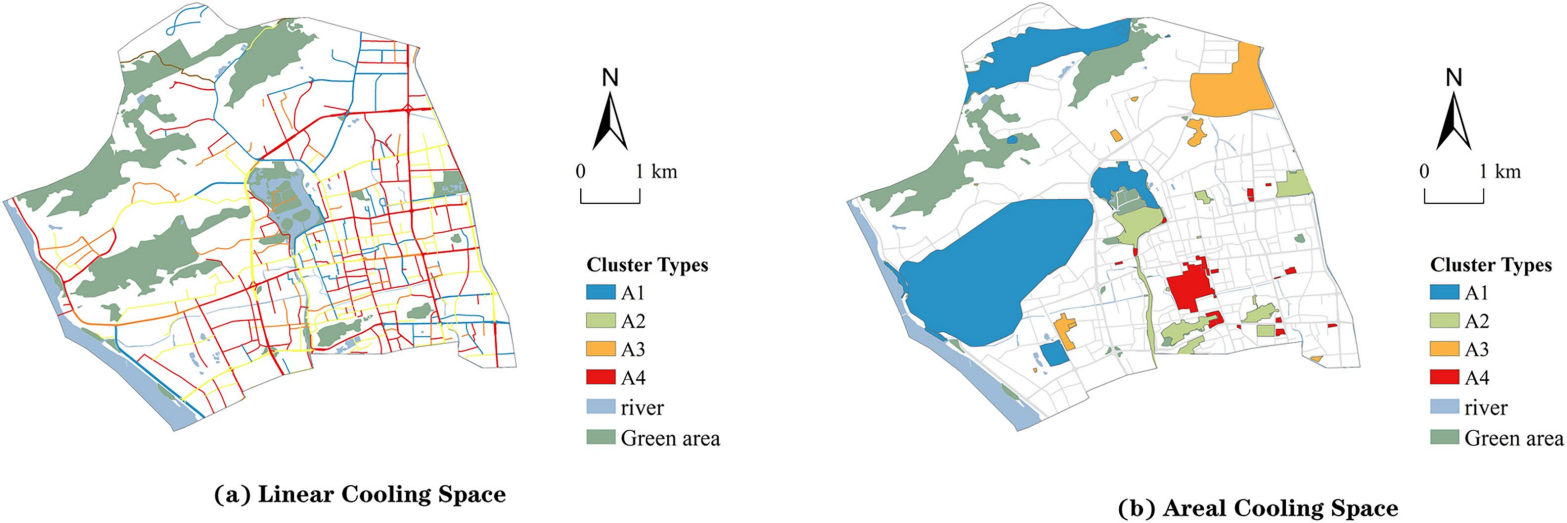
Visualization of cluster analysis results. **(a)** Cluster types for linear cooling spaces. **(b)** Cluster types for areal cooling spaces.

Further cluster analysis divided the linear cooling spaces into four categories (A1–A4), each exhibiting distinct justice deficit characteristics (Figure 7a). Category A1 corresponds to Distribution and Recognition Deficit Spaces, Category A2 to Distributive Justice Deficit Spaces, Category A3 to Justice-Balanced Spaces, and Category A4 to Systemic Justice Deficit Spaces (Table 8). Areal cooling spaces were also classified into four types (B1–B4), including All-Dimension Justice Fulfilled Spaces, Recognition Justice Deficit Spaces, Distributive Justice Deficit Spaces, and Systemic Justice Deficit Spaces (Figure 7b). Variance analysis confirmed that inter-category differences were statistically significant for both linear and areal spaces (*p* < 0.001), validating the robustness of the clustering results (Table 9).

**Table 8:** Linear cooling space cluster analysis results.

Dimension	Type			
	Distribution & recognition deficit space (A1)	Distributive justice deficit space (A2)	Justice-balanced space (A3)	Systemic justice deficit space (A4)
Distributive justice dimension	0.28	0.362	0.408	0.279
Recognition justice dimension	0.213	0.733	0.425	0.339
Procedural justice dimension	0.946	0.938	0.934	0.429
Count of spaces	108	84	76	19
Total	279			

**Table 9:** Areal cooling space cluster analysis results.

Dimension	Type			
	All-dimension justice fulfilled space (B1)	Recognition justice deficit space (B2)	Distributive justice deficit space (B3)	Systemic justice deficit space (B4)
Distributive justice dimension	0.874	0.721	0.457	0.208
Recognition justice dimension	0.894	0.372	0.754	0.192
Procedural justice dimension	0.912	0.661	0.747	0.355
Count of spaces	13	11	11	19
Total	54			

## 4 Discussion

### 4.1 Comparison with previous studies

Our findings identify high-density built-up areas, aging neighborhoods, and commercial cores as major hotspots of climate injustice. This result is consistent with a broad body of research linking compact urban morphology, restricted ventilation, and insufficient greenery to amplified thermal stress (20, 48, 49). We also found that socioeconomic vulnerability and uneven access to resources exacerbate inequitable exposure. This finding is consistent with evidence reported in urban heat studies in both Asian and Western contexts (50, 51). However, unlike previous macro-scale assessments (20, 21), our micro-scale, justice-oriented approach reveals significant intra-urban heterogeneity within these known risk zones.

This study’s primary contribution lies in its micro-scale insights. We found that large commercial plazas and open squares (e.g., The Mixc) exhibit pronounced distributive justice deficits, despite being highly accessible. This is largely due to extensive impervious surfaces and a lack of shading. This finding aligns with microclimate research demonstrating that open plazas often accumulate extreme heat and suffer from poor pedestrian usability (52, 53). This directly challenges the common assumption that the mere presence of open or green space automatically enhances thermal comfort.

Furthermore, this study distinguishes between linear and areal cooling spaces, whereas previous studies often treat public or green spaces as homogeneous environments (54, 55). This distinction is critical. We found that linear cooling spaces are more strongly influenced by morphological parameters that govern heat exposure, such as height-to-width ratio, Sky View Factor, and orientation. In contrast, areal cooling spaces show patterns more closely related to governance and social accessibility. This latter finding supports research that emphasizes procedural and recognition justice as key dimensions of equitable climate adaptation (56, 57).

### 4.2 Driving mechanisms of climate injustice

The identified patterns of climate injustice are not randomly distributed but are driven by underlying urban development trajectories and governance processes. The findings of this study point to three key mechanisms shaping these inequities.

(1) Historical path dependency explains the persistent vulnerability of old urban districts. The clustering of Systemic Justice Deficit Spaces (A4, B4) in traditional neighborhoods such as Sanfang Qixiang reflects the inertia of past planning. Narrow streets, low sky view factors, and limited greenery have been structurally locked in, and these morphological constraints now compound with demographic aging to intensify risks (58).
(2) Market-driven urban renewal generates new forms of climate injustice. In contrast to historical vulnerabilities, the distributive deficits (B3) observed in modern commercial centers such as The Mixc stem from design priorities that favor commercial esthetics and accessibility over thermal comfort. This reflects broader climate gentrification trends, where climate-resilient green infrastructure concentrates in affluent areas, while public and peripheral spaces face inadequate adaptation (59, 60).
(3) Governance and procedural failures directly produce a spatial mismatch between resource supply and population needs. Peripheral zones with poor access to medical and cooling facilities illustrate a top-down, supply-oriented model that neglects vulnerable groups and limits community participation. The resulting lack of procedural justice reinforces both recognition and distributive inequities in a self-reinforcing cycle (61).

### 4.3 Governance strategies for climate justice: differentiated interventions for linear and areal spaces

Outdoor cooling spaces in Gulou District exhibit significant disparities across the three dimensions of distributive, recognition, and procedural justice. These disparities directly affect residents’ capacity to respond to extreme heat and their access to adaptive resources. To address the uneven distribution of climate vulnerability, this study applies the K-means clustering method to identify representative high-risk types in both linear and areal cooling spaces. Based on these findings, targeted governance pathways for promoting climate justice are proposed.

In linear cooling spaces (62, 63), Distributive Justice Deficit Spaces (A2) exhibit weaknesses in the distributive dimension but perform relatively well in recognition and procedural justice. These streets demonstrate some level of participation mechanisms and sensitivity to vulnerable groups, yet the allocation of cooling resources remains insufficient. A2 spaces are widely distributed in the western, central, and southeastern parts of Gulou District, commonly occurring along main roads, transitional zones, and older alleyways. They represent a concentrated manifestation of resource shortages. Priority interventions should focus on improving shading, ventilation, and microclimate regulation facilities, while embedding procedural mechanisms such as resident participation and community co-construction (64). Justice-Balanced Spaces (A3) perform evenly across all three dimensions, representing a relatively ideal type of cooling street. They combine adequate resource supply with responsiveness to community needs and compliance with procedural norms. These spaces a are mainly located in central and eastern districts, particularly in mixed residential–commercial areas and community corridors. They should be prioritized for inclusion in systematic upgrading programs, such as ecological streets and healthy pathways, to strengthen climate justice demonstration and institutional integration. Systemic Justice Deficit Spaces (A4) score low across all three dimensions, reflecting resource scarcity, marginalized populations, and weak governance participation. A4 spaces are primarily concentrated at the edges of central districts and in high-density southern areas. They are characterized by enclosed environments, poor ventilation, and limited greenery, forming blind spots in urban climate risk governance. High-priority interventions should include the opening of green corridors, functional redistribution, intelligent monitoring, and targeted services for vulnerable groups to enhance overall climate justice.

In areal cooling spaces, All-Dimension Justice Fulfilled Spaces (B1) achieved high scores across all three dimensions of justice, characterized by equitable resource distribution, strong recognition of social groups, and transparent governance. These spaces are mainly located in the western and northern parts of the city, concentrated in large open green areas and lakeside parks, such as Jinniu Mountain Park and West Lake Park. With abundant environmental resources and service capacity, they should be prioritized as high-quality cooling spaces for protection. Recognition Justice Deficit Spaces (B2) perform well in distributive and procedural justice but score considerably lower in recognition justice. This indicates that, despite relatively favorable environmental conditions, the needs of vulnerable groups are not sufficiently considered. Such spaces are primarily distributed around old residential communities and include areas near West Lake Park and Yushan Scenic Park. Targeted interventions should focus on enhancing attention to heat-vulnerable populations such as the old adult and children through inclusive design and service provision. Distributive Justice Deficit Spaces (B3) are defined by low scores in the distributive dimension, reflecting insufficient allocation of physical environmental resources such as shading and ventilation. These spaces are typically located in peripheral built-up areas, including squares and exposed plots, such as The Mixc and the Sports Center. With substantial heat loads, they require interventions such as supplementary greening and facility retrofitting to improve climate adaptation (65). Systemic Justice Deficit Spaces (B4) scored low across all three justice dimensions, representing the most severe form of climate justice deficit. These spaces are often found in old neighborhoods and commercial cores, such as Three Lanes and Seven Alleys, and are characterized by high population demand, intense environmental stress, and weak governance capacity. They should be treated as priority areas for intervention. Policies should focus on establishing cooling service networks, enhancing medical response capacity, and strengthening social support mechanisms (66). This would prevent vulnerable groups from being marginalized under extreme heat conditions and reflect the principle of distributive justice: “the more vulnerable, the higher the priority” (67).

### 4.4 Advantages and contributions of the assessment framework

This study reconstructs the traditional “Exposure–Sensitivity–Adaptive Capacity” (VSD) model by embedding justice dimensions into the indicator framework to develop a multi-source system for assessing climate justice. In the distributive justice dimension, attention is given to the equitable allocation of heat-mitigation resources across spatial units. This assessment is spatially specific: for linear spaces, the framework uses Sky View Factor (SVF), street enclosure, Green View Index (GVI), and Land Surface Temperature (LST) to capture heat exposure, reflecting both accumulation and shading potential. For areal spaces, it employs vegetation cover and LST to represent microclimatic regulation capacity. In the recognition justice dimension, population aggregation, the share of sensitive groups, and housing prices identify the spatial distribution of heat-vulnerable populations, revealing how social inequality shapes exposure. In the procedural justice dimension, the number of indoor cooling facilities and medical institutions measures the fairness of public service provision and access to emergency support.

At the evaluation scale, micro-level units such as streets, squares, and green areas were adopted, extending the applicability of the VSD framework to urban micro-spaces and public domains. By integrating K-means clustering with analysis of variance (ANOVA), a typology of spatial classifications and justice-deficit categories was developed. These results provide a graded basis for targeted policy interventions and spatial governance. Moreover, this study broadens the practical application of climate justice assessment at the urban spatial scale, offering both theoretical support and empirical validation for the localized adaptation of assessment methods.

### 4.5 Research limitations

This study has several limitations that warrant further refinement in future research. First, the evaluation relied on a static indicator system and did not fully account for the temporal dynamics of human behavior. Heat risk is inherently time-dependent, yet the current indicators are derived from single time points or averaged states, which cannot capture the dynamic relationships between exposure and health impacts across different periods. Future studies could incorporate time-series indicators, such as real-time population mobility from mobile phone data or temporal analyses of heat-related content on social media, to better reveal the spatiotemporal variability of heat risk. Second, the subjective dimension of thermal perception remains insufficiently addressed. The present framework is largely based on objective physical and demographic data, but it fails to reflect the lived experiences and challenges of vulnerable groups such as outdoor workers, children, and the old adults under extreme heat. Future work could integrate approaches such as social media text mining, questionnaire surveys, and emotion recognition from street-view images to enrich indicators of perceived heat stress and adaptive behavior.

## 5 Conclusion

This study developed a climate justice assessment framework for urban outdoor cooling spaces based on heat vulnerability, integrating the three dimensions of distributive, recognition, and procedural justice. An empirical analysis was conducted for both linear and areal cooling spaces in Gulou District, Fuzhou. The main findings are as follows:

(1) The assessment results reveal pronounced spatial inequities in the capacity of cooling spaces to withstand heat risks. In linear cooling spaces, 37.18% showed low justice levels, mainly in the southern and southeastern parts of the district and along major traffic corridors. These areas bear heavy environmental loads, dense populations, and limited greening. In areal cooling spaces, 44.45% were categorized as unjust, primarily located in central and eastern built-up areas, historic neighborhoods, and public activity hubs, such as the Dongjiekou business district, Wuyi Square, and the dense eastern urban core.
(2) K-means clustering further classified cooling spaces into four vulnerability types. Linear spaces were grouped as Distribution & Recognition Justice Deficit (35.84%), Distributive Justice Deficit (30.11%), Justice-Balanced (27.24%), and Systemic Justice Deficit (6.81%). Areal spaces were categorized as All-Dimension Justice Fulfilled (24.07%), Recognition Justice Deficit (20.37%), Distributive Justice Deficit (20.37%), and Systemic Justice Deficit (35.19%).
(3) The analysis revealed a spatial mismatch between adaptive infrastructure and vulnerable populations. Areas with better facilities do not necessarily coincide with high-risk or low-income neighborhoods, reinforcing intergenerational and group-based inequalities. This finding highlights the need for equitable resource allocation and inclusive governance to support vulnerable groups under heat stress.

Overall, this study introduces a quantifiable framework for integrating climate justice into urban heat risk governance. Future research could incorporate dynamic indicators of thermal stress and social perception data to enhance temporal sensitivity and responsiveness, thereby providing sustainable support for urban health resilience planning under intensifying heatwave conditions.

## Data Availability

The datasets presented in this article are not readily available because the datasets generated for this study are not publicly available because they contain location-based or personally sensitive information. Data may be available from the corresponding author upon reasonable request and with institutional approval. Requests to access the datasets should be directed to yangjs@fjut.edu.cn.
