# Correction: A VSD-based framework for assessing climate justice in urban outdoor cooling spaces: a case study of Fuzhou, China

**DOI:** 10.3389/fpubh.2025.1766893

**Published:** 2026-01-12

**Authors:** Fengxiao Cao, Yimeng Zhou, Yu Luo, Yuming Shang, Jinsu Yang, Di Yang

**Affiliations:** 1 College of Architecture and Planning, Fujian University of Technology, Fuzhou, China; 2 Key Laboratory of New Technology for Construction of Cities in Mountain Area, Ministry of Education, Chongqing University, Chongqing, China

**Keywords:** climate justice, heat vulnerability, urban outdoor cooling spaces, evaluation and optimization, VSD framework

In the published article, there was an error in the affiliation list. Affiliation 2 “College of Architecture and Urban Planning, Fujian University of Technology, Fuzhou, China” was erroneously included as a duplicate of Affiliation 1. The affiliations have been consolidated, and the subsequent affiliation has been renumbered. Consequently, the affiliation reference for author Jinsu Yang has been corrected from 2, 3 to 1, 2.

The original version of this article has been updated.

