# Supplementary material for: A VSD-based framework for assessing climate justice in urban outdoor cooling spaces: a case study of Fuzhou, China
Source: Front Public Health. 2025 Dec 12;13:1724719. doi: 10.3389/fpubh.2025.1724719 (PMC12741077; doi:10.3389/fpubh.2025.1724719)
Supplement: Supplementary file 1 [file Table_1.pdf]

# *Supplementary Material*

**Supplementary Table 1. Index system for climate justice assessment**

| Target Layer | Space Type | Guideline Dimension | Index Layer | Indicator Weight | Data Acquisition and Calculation Method |
|---|---|---|---|---|---|
| Assessment of Climate Justice in Outdoor Cooling Spaces | Linear Cooling Spaces（A1） | Distributive Justice（B1） | Sky View Factor （C1） | 0.190 | Sampling points were placed along streets to calculate the sky view factor (SVF). The mean value was derived and assigned to each street using zonal statistics. |
| | | | Street Canyon Ratio （C2） | 0.550 | Building heights (H) on both sides of the street and street width (W) were extracted to calculate the H/W ratio. The mean value was assigned to each street using zonal statistics. |
| | | | Green View Index （C3） | 0.131 | Street view images were collected along streets to calculate the green view index (GVI). The mean value was assigned to each street using zonal statistics. |
| | | | Surface Temperature（C4） | 0.129 | A 50 m buffer was generated for each street. Land surface temperature (LST) grids within the buffer were extracted, and the mean value was assigned to the street. |

| | | | | |
|---|---|---|---|---|
| | Recognition Justice（B2） | Population Aggregation（C5） | 0.187 | A 50 m buffer was generated for each street. Population heatmap or mobile signaling data within the buffer were used to calculate population density, which was assigned to the street. |
| | | Sensitive Population（C6） | 0.527 | A 50 m buffer was generated for each street. Census block data were overlaid to calculate the proportion of sensitive populations, which was assigned to the street. |
| | | Housing Prices（C7） | 0.286 | A 50 m buffer was generated for each street. Residential POIs within the buffer were used to calculate average housing prices, which were assigned to the street. |
| | Procedural Justice（B1） | Indoor Cooling Facilities（C8） | 0.623 | A 50 m buffer was generated for each street. Indoor cooling facility POIs within the buffer were counted to calculate facility density, which was assigned to the street. |
| | | Medical Facilities（C9） | 0.377 | A 50 m buffer was generated for each street. Medical facility POIs within the buffer were counted to calculate facility density, which was assigned to the street. |
| Areal Cooling Space（A2） | Distributive Justice（B1） | Vegetation Coverage（C10） | 0.648 | A 500 m buffer was generated for parks and squares. NDVI grids within the buffer were used to calculate the mean value, which was assigned to the areal feature. |

| | | | |
|---|---|---|---|
| | Surface Temperature（C11） | 0.352 | A 500 m buffer was generated for parks and squares. Land surface temperature (LST) grids within the buffer were used to calculate the mean value, which was assigned to the areal feature. |
| Recognition Justice（B1） | Population Aggregation（C12） | 0.390 | A 500 m buffer was generated for areal features. Population heatmap data within the buffer were used to calculate the mean value, which was assigned to the areal feature. |
| | Sensitive Population（C13） | 0.399 | A 500 m buffer was generated for areal features. Census block data were overlaid, and the proportion of sensitive populations was calculated using area-weighted averaging, which was assigned to the areal feature. |
| | Housing Prices（C14） | 0.211 | A 500 m buffer was generated for areal features. Residential POIs within the buffer were used to calculate average housing prices, which were assigned to the areal feature. |
| Procedural Justice（B1） | Indoor Cooling Facilities（C15） | 0.533 | A 500 m buffer was generated for areal features. POIs such as shopping malls and libraries within the buffer were counted, and the results were assigned to the areal feature. |

| | | | |
|---|---|---|---|
| | Medical Facilities（C16） | 0.467 | A 500 m buffer was generated for areal features. Medical facility POIs within the buffer were counted, and the results were assigned to the areal feature. |
